# Nurses’ Experiences of Futile Care at Intensive Care Units: A Phenomenological Study

**DOI:** 10.5539/gjhs.v7n4p235

**Published:** 2015-01-14

**Authors:** Leili Yekefallah, Tahereh Ashktorab, Houman Manoochehri, Alavi Majd Hamid

**Affiliations:** 1 International Branch, Shahid Beheshti University of Medical Sciences, Tehran, Iran; 2 Faculty of Nursing and Midwifery, Shahid Beheshti University of Medical Sciences, Tehran, Iran; 3 Faculty of Biostatistics, Shahid Beheshti University of Medical Sciences, Tehran, Iran

**Keywords:** futile care, nurse experience, Intensive Care Unit, phenomenological study, qualitative research

## Abstract

The concept and meaning of futile care depends on the existing culture, values, religion, beliefs, medical achievements and emotional status of a country. We aimed to define the concept of futile care in the viewpoints of nurses working in intensive care units (ICUs). In this phenomenological study, the experiences of 25 nurses were explored in 11 teaching hospitals affiliated to Social Security Organization in Ghazvin province in the northwest of Iran. Personal interviews and observations were used for data collection. All interviews were recorded as well as transcribed and codes, subthemes and themes were extracted using Van Manen’s analysis method. Initially, 191 codes were extracted. During data analysis and comparison, the codes were reduced to 178. Ultimately, 9 sub-themes and four themes emerged: uselessness, waste of resources, torment, and aspects of futility. Nurses defined futile care as “useless, ineffective care giving with wastage of resources and torment of both patients and nurses having nursing and medical aspects”

As nurses play a key role in managing futile care, being aware of their experiences in this regard could be the initial operational step for providing useful care as well as educational programs in ICUs. Moreover, the results of this study could help nursing managers adopt supportive approaches to reduce the amount of futile care which could in turn resolve some of the complications nurses face at these wards such as burnout, ethical conflicts, and leave.

## 1. Introduction

From various aspects, care giving is the main principle and essence of nursing and theorists in this field have frequently correlated health with care giving (Biley, 2005). Taylor (2005) believes that care giving is actually different from treatment and can be done either directly or indirectly, which includes nurturing, some professional activities, and some crucial decision making. Care giving means providing nursing services spiritually, physically and mentally to both patients and their families (Taylor, Lillis, & Lemone, 2005). the goal of care giving is to give hope to the patients for better recovery in the future (Potter & Perry, 2009).

Currently, care is not given efficiently in clinical settings (Miyawaki, 2010) due to increased human experiences and interactions regarding end-of-life care with the help of technological interventions especially in intensive care units (ICUs). Although technology has increased life expectancy, it has not enhanced people’s quality of life (Heland, 2006). In the US, at least one out of every five patient admitted to the ICU dies (Espinosa, Anne, Lene, Brenda, & Teresa, 2010). Nearly half of the patients who die in the US hospitals are admitted to ICUs and receive futile care.

Futile care is defined as medical care with therapeutic effects that is futile and useless for the patient. The term “futile care” was first defined in 1980 and used in the texts of medical ethics in 1990 (Gabbay, Calvo-Broce, Meyer, Trikalinos, Cohen, & Kent, 2010). The definition of futile care differs based on the patient’s condition and the nurse’s personal values (Palda, Bowman, McLean, & Chapman, 2005). Some nurses define futile care based on the possibility of the patient’s survival while others define it in terms of patient’s quality of life after survival (Heland, 2006). Moreover, the definition of futile care depends on how people perceive the quality or quantity of life, ethical beliefs, and judgment regarding successful or unsuccessful treatment. Although several definitions have been proposed for futile care, there is no consensus on its definition. Its definition varies depending on the patients values and beliefs about the limitations of treatment and the patients’ and their families’ level of information. It should be noted that futile care does not only include end-of-life care but also any care that does not lead to patient’s survival as well as discharge or maintain and enhance his/her quality of life (Chwang, 2009).

The costs of futile care add up to 30% of all treatment costs. The lowest and highest costs of end-of-life care in the ICUs of educational hospitals are between $53,432 and $93,000 to $105,000 per patient. Futile care is provided for all patients regardless of disease severity. Studies have shown that, without futile care, millions of dollars would be saved in the salaries of physicians (Neuberg, 2009).

In the ICUs, a considerable proportion of resources are allocated to futile care (Niderman & Berger, 2010). Researchers have found that 40-60% of care given in the ICUs is futile (Palda et al., 2005). Studies show that futile care increases risks, losses as well as costs and creates painful complications such as bedsores, catheter-induced infections and ventilator-induced pneumonia (Neuberg, 2009).

Therefore, plans to enhance the quality of nursing care and accessibility to intensive care consultants is necessary for policy making regarding the conditions of futile care (Palda et al., 2005). Nurses play a key role in futile care or its prevention. Previous experiences with such situations could help nurses have important and positive effects on patients and their families.

In Iran, futile care is also a challenge for nurses in ICUs. However, despite the prevalence and complications of issues related to futile care among nurses working in ICUs, few studies have been performed on the relationship between nurses and patients as well as their families during futile care (Heland, 2006). To identify and analyze these challenges, it is initially necessary to understand the nurses’ experiences at ICUs in provision of futile care. This preliminary step is important for presenting solutions and approaches for enhancing the quality of nursing care in these wards (Espinosa et al., 2010). Therefore, we aimed to assess the nurses’ experiences of futile care in ICUs.

## 2. Method

### 2.1 Study Design

This phenomenological study was conducted to understand the experiences of nurses working in ICUs with respect to the concept of futile care.

### 2.2 Setting and Sample

The statistical population of this study included nurses working in the ICUs of 11 teaching hospitals and hospitals affiliated to Social Security organization in Qazvin province in the northwest of Iran. 25 Icu nurses were selected for personal interviews using the purposive sampling method. Icu Nurses with at least BS degree and one-year experience with normal auditory and verbal function were included in this study.

### 2.3 Ethical Consideration

research ethics approval was obtained from Shahid Beheshti Medical Sciences University research ethics Board in Tehran. It should be stated that ethical considerations such as data confidentiality were met at all stages of the study.

### 2.4 Data Collection

data were collected using semi-structured interviews and non-structured observations until data saturation. All interviews were transcribed verbatim. The interviews lasted from May to November 2013. In order to start the interviews and reach a comprehensive understanding of the phenomenon, the participants were asked to express their experiences of futile care. The subsequent questions were asked on the basis of participants’ description of futile care. Sample questions are as follows: What does futile nursing care mean? Describe your experiences of futile care. What measures do you often label as futile?

The durations of the interviews were 30-86 minutes over 1-3 sessions. All interviews were performed by first author. However, the interviews were analyzed by all authors. After each interview, data were quickly transcribed and reviewed word by word for several times to increase accuracy and understanding. From this point, the transcribed data were the raw source for defining the concept of futile care in this study.

Non-structured observations were also used during semi-structured interviews for recording the participants’ behavior, non-verbal communication, appearance, facial expression, and eye contact.

### 2.5 Data Analysis

Data were analyzed based on Van Manen’s phenomenological method. Van Manen (2001) introduced the following six activities as the operational approach for hermeneutic phenomenology: (Van Manen, 2001).

1) Paying attention to the nature of the lived experience, the first stage in study is notification to nature of the lived experience.
  The tendency and interest of the first author to study the phenomenon of futile care in ICUs dates back to the time when she worked as a supervisor at a hospital. During ICU rounds, she noticed that some nurses considered some procedures futile. They gave futile care for specific reasons such as fear of supervision. Also, it seemed that such care affected their performance and attitude. Therefore, the author became interested to study futile care and its related factors.
2) Discovering a specific experience as lived:
  At this stage, the researcher studies an experience exactly as it is lived and not the way it is conceptualized. In our study, the main data collection method was interviews with open-ended questions. The interviews started with a simple question to begin conversation and continued with general questions preferably focusing on targeted issues.
3) Contemplation on essential themes that define the phenomenon’s characteristics:
  Three approaches have been used for clarifying and uncovering thematic aspects of a phenomenon: holistic, selective or labeling, and detailed or line by line. We used the detailed and selective approaches for separating thematic sentences. Initially, each interview was read several times and we asked ourselves: Which statements were necessary for describing the phenomenon or experience. Then, the statements were identified and underlined and the statements similar to the patients’ words (descriptive) or their meanings and interpretations (interpretive) were written. By merging and categorizing thematic sentences, major themes and minor categories were obtained.
4) Describing the phenomenon using the art of writing and rewriting:
  In this stage, the authors organized the written descriptions about the participants’ statements and presented examples of what they had said.
5) Establishing and maintaining a strong and oriented relationship with the phenomenon:
  The researchers tried to constantly review the main research question at all stages including data analysis and theme extraction. In all stages of data analysis, the researchers initially reviewed the main research question and tried to extract themes according to the main research question.
6) Balancing the Research Context by Considering Parts and Whole:
  By using a selective and detailed approach, the researchers defined the concept of futile care and its related factors in Iran. For thematic analysis, frequent inductions and deductions were used considering the research question.

During coding process: initially, each interview was read several times and we asked ourselves: which statements were necessary for describing the phenomenon or experience. Then, the statements were identified and underlined and the statements similar to the patients’ words (descriptive) or their meanings and interpretations (interpretive) were written. In all stages of data analysis, the researchers initially reviewed the main research question and tried to extract themes according to the main research question. At the end stage by merging and categorizing thematic sentences, major themes and minor categories were obtained. In initial coding, 191 codes were extracted. During data reduction, the codes were reduced to 178. Ultimately, five categories, 4 themes (uselessness, waste of resources, torment, and aspects of futility), and nine subthemes were emerged.

Trustworthiness: the accuracy of qualitative findings was determined using validity, verifiability, reliability, and transferability. To increase acceptance, the researchers had adequate and close interactions with the participants. The interview files and extracted codes were reviewed by external observers and their complementary opinions were considered. Verifiability was confirmed by external nurses and supervisors.

## 3. Results

In the present study, 21 women and 4 men were enrolled between 27-45 years with mean experience of 10.24 years (range: 1-20 years). The mean work experience of the participants at ICUs was 7.02 years (range: 1-15 years). The participants’ demographic characteristics are shown in Table 1.

**Table 1:** Demographic characteristics of the participants (n=25)

Characteristics		N (%)
**Sex**	Female	21 (84)
	Male	4 (16)
**Marital status**	Single	13 (52)
	Married	12 (48)
**Educational status**	BSc	22 (88)
	MSc	3 (12)
**Position**	Head nurse	2 (8)
	Staff nurse	2 (8)
	In rotation nurse	21 (84)
**Type of ICU**	Surgical	6 (24)
	Internal	8 (32)
	Cardiac surgery	3 (12)
	General	5 (20)
	Trauma	3 (12)

Note: BS=Bachelor of Science; MS =Master of Science.

After data analysis, five categories, 4 themes (uselessness, waste of resources, torment, and aspects of futility), and nine subthemes were emerged. The participants defined futile care as “useless and ineffective care associated with waste of resources and torments of patients and nurses with nursing and medical aspects”. The different aspects of futile care include measures taken by medical team such as futile admission, futile diagnostic procedures, futile medical instructions, and measures taken by the nursing team such as: futile nursing interventions and irrelevant duties to nursing. These themes and subthemes are shown in Table 2.

**Table 2:** Theme and Sub-theme of futile care

Theme	Sub-theme	
Uselessness	Aimlessness of care
	Ineffective care		
	Certainty in lack of recovery
Waste of resources	Waste of time	
	Waste of money
Torment	Patients’ suffering		
	Nurses’ suffering	Categories	

Aspects of futility	Measures taken by the medical team	Futile admission
		Futile diagnostic procedures
		Futile medical orders
	Measures taken by the nursing team	Futile nursing interventions
		Irrelevant duties for nurses

1). “Uselessness” was the first emerging theme of this study. The subthemes of this theme included aimlessness of care, ineffective care, and certainty of the patient’s lack of recovery.

1). “Uselessness” was the first emerging theme of this study. The subthemes of this theme included aimlessness of care, ineffective care, and certainty of the patient’s lack of recovery.

*Aimlessness of Care*

Many nurses believed that futile care is aimless and not effective in improving the patients’ health. The nurses stated that effective care meets the patients’ physical, mental, emotional, social, and spiritual needs. One of the experiences stated by the participants about this sub-theme was as follows:

“*Futile care means doing things for the patient that are useless; things, you know, that have no effect on patient’s recovery…I mean, he might not get better and we are just doing things just to support to pass these final days.”* (P16)

Ineffective Care

Some nurses stated that effective care is one that leads to patient’s recovery. Other nurses contented that effective care is one that has the least harm and the highest benefit for the patients. They believed that effective care has least complications and is ultimately goal-oriented and lifesaving for the patients. One of the participants said, *“Ineffective care means you are doing things that do not make the patient any better, the patient is unresponsive to the things you do…; it is an awful feeling. You are somehow sure he is dying and no one can do a thing.”* (P18)

*Certainty in Lack of Recovery*

The participants stated that caring for end-stage patients is futile. One nurse said, “*When we really know we are dealing with an end-stage patient and one who is dying, our care is futile. For example, a 90-year-old patient with colon cancer is a no-code patient who is left here to expire and our care is useless. For such a patient, even if we perform CPR, we would break her ribs. Even if CPR is successful, we are making her suffer. In such cases, I call the doctor and ask him whether we should perform CPR for a patient in case of arrest and he says there is no need.”* (P2)

2). “Waste of resources” was the second theme that emerged in our study. This theme included two subthemes: waste of time and waste of money.

*Waste of Time*

Most nurses believed that futile care reduces the quality of nursing care due to the lack of time that could otherwise be used for more useful work. One nurse said: “*Working with futile cases takes so much time…. Suction, chest-physio, and medications take time, which could be used more effectively for other patients.”* (P3)

*Waste of Money*

The admission of futile patients is a burden on the entire system and their families. In this regard, one nurse said, *“So much money is wasted because of what we do for futile patients although we know they would not survive. So many tests are written for them. It really hurts when all this work and money are wasted…since sometimes their death is delayed because of what we do.”* (P3)

3). “Torment” was the third emerging theme in our study. The subthemes of torment included “patients’ suffering” and “nurses’ suffering”.

*Patients’ Suffering*

One of the ethical principles nurses obliged to obey is not to harm the patients. One nurse stated, *“Sometimes, we feel the patients are really suffering and when they die, we can see peace and calmness in their faces. When we could avoid invasive procedures, why shouldn’t we let the patient die? We know the patient is really suffering.”* (P18)

*Nurses’ Suffering*

The participants claimed that the patients’ suffering during invasive procedures was also painful for them. For example, one nurse expressed, *“Sometimes, I get really upset when I take blood samples from them, especially arterial blood samples. I wish we didn’t have to. I even told one of the doctors to cut down the orders for sampling. For example, we had a patient for whom BS had to be checked four times a day. We didn’t even have a Glucometer or any kit and had to send the blood samples to the lab. We also had to take arterial blood sample.”* (P13)

4). “Aspects of futility” was the fourth extracted theme. The subthemes of this theme included measures taken by the medical team (futile medical orders, futile diagnostic procedures, and futile admission) and measures taken by the nursing team (futile nursing interventions and irrelevant duties of nursing).

*Futile Medical Orders*

The participants stated that orders interfered with each other, various complementary orders, and lack of attention to the results of orders were futile and, ultimately, tired the personnel and wasted their time. Moreover, they stated that prescribing expensive drugs and different types of tests for a dying patient was useless. One nurse said, *“For example, last week, we had a patient we knew would expire. The doctor ordered a bone marrow for him but his family did not give consent. Afterwards, the doctor spoke to them and asked them whether they wanted to know what the reason for the death of their father was. Finally, they agreed.”* (P11)

*Futile Diagnostic Procedures*

The participants stated that some physicians opted to perform various invasive diagnostic procedures without notifying patients and their families. One nurse said: *“The doctor orders pleural fluid tap, lumbar puncture and many other things with no logical reason while he knows the patient is going to die and still orders an emergency sonography or endoscopy. This really tires the nursing team and decreases their motivation. Believe me! The nurses on duty are too tired to walk because of all these orders for a GCS-3 patient.”* (P13)

*Futile Admission*

The participants stated that, unfortunately, some physicians admit patients to ICUs with no reasonable justification. They are either patients who can be admitted to other wards or dying patients whom nothing can be done for. One nurse said, *“Some doctors tell the family of, for example, a GCS-3 patient to bring him to hospital for surgery; therefore, the family won’t listen to us saying it is useless….”* (P8)

*Futile Nursing Interventions*

The participants stated that, unfortunately, 80% of ICU care is futile. Antibiotic therapy, routine lab tests, injection of blood products and caring for patients with no real need to be admitted a ICUs are among futile nursing interventions mentioned by the participants.

Participant 12: *“For example, 2-hour vital sign checking when I know nothing has happened to the patient during this time or having a useless ABG for a patient”*

Participant 11: *“At the end of a shift, some nurses avoid heavy workload and a dying patient has so much work to do; therefore, they inject atropine to pass the patient to the next-shift nurses. Because the patient has been admitted for a long time and we should do all secretarial work of the unit.”*

*Irrelevant Duties for Nurses*

Unclear job description of nurses prevents optimal performance, reduces productivity and increases futile care. Using ICU nurses as secretaries and making them purchase prescriptions and count medications are among the items mentioned by the participants in this subtheme.

Participant 15: *“It is a pity that an ICU nurse should sit, count and enter drugs into the computer system. For example, I have to sit and enter drugs into our computer system the day after a night shift; one hour of me is wasted and these are all useless work.”*

## 4. Discussion

This research aimed to define the concept of futile care. We found that nurses defined futile care as “useless, ineffective care giving with wastage of resources and torment of both patients and nurses having nursing and medical aspects”. The differences between this and previous definitions could be attributed to the differences in study populations.

Various studies have shown that one of the characteristics of high-quality nursing care is being goal-oriented. Goal-oriented care is the most desirable one based on the patients’ needs as well as treatment goals (Pazarghadi 2007). We found that, in the nurses’ perspectives, futile care does not improve the condition of patients and is provided aimlessly. Most nurses believed that working with purpose increases the quality of care giving because it is evaluated based on its goal. Smith (2012) found that a procedure or care is considered futile when its aim is inaccessible or impossible (Smith, 2012).

Considering that any procedure has some level of known or unknown complications, the patient should only be exposed to such complications when some degree of benefit is perceived for the patient. However, in futile procedures, the patients suffer from these complications without any improvement. Some complications such as antibiotic resistance might pose some risks for the society as well as impose a financial burden on families and the community. Therefore, inflicting unnecessary pain, suffering and side effects on patients is unethical (Aramesh 2008). In one study, 50% of nurses and 70% of home-based caregivers reported that care provided to the patients were against the standards and led to the caregivers’ moral distress (Meltzer & Huckabay, 2004). Sibbald (2007) found that many of the participants described futile care as pain and suffering and claimed that it would only prolong a painful life. Most descriptions denote that it takes a considerable amount of resources without significant effect. The consumption of considerable resources without any hope for patients’ recovery, their partial independence, and their interaction with the environment were also included in the descriptions of futile care by the participants (Sibbald, 2007).

Sometimes nurses face patients that spend a great amount of money for admission to the ICU. However, staying at ICU does not have any benefit for them and, in many cases, all or part of medical costs is covered by public resources such as insurance. Futile procedures are, in fact, a waste of such resources. Moreover, the physician is responsible for preventing excess costs. On the other hand, the physicians’ time and diagnostic and pharmaceutical facilities that are used in a futile manner are also medical resources. Using such resources for futile treatments not only wastes money but also delays accessibility to these resources for patients who really need them (Aramesh, 2008).

One of the nurses challenges face with respect to futile care is related to CRP and weak management in no-resuscitation order. Implementing a decision-making plan and strengthening the nurse’s role in guiding the families of patients during this process is a challenge most ICUs face (Heland, 2006). The nurses stated that performing CRP for some patients was really useless, but according to national laws in Iran all “no-code” patients must be resuscitated. Performing CRP on a patient with end-stage cancer or incurable diseases only increases their pain and suffering and this is in conflict with ethical principles such as the patient’s will to accept the type of treatment (Atashzadeh Shoorideh, 2011).

Some aspects of futile care included measures taken by the medical (futile admission, diagnostic procedures, and medical orders) and nursing (futile nursing interventions and irrelevant duties for nurses) team. Futile care is increased when physicians do not pay attention to the patients’ and their families’ desires and do not provide insight for them. Some participants believed that most routine procedures are not only futile but impose some risks for the patients. They stated that many routine procedures should be reconsidered because they harm patients. Such routine procedures are part of the caregiver’s responsibilities. Therefore, the nurses felt responsible for performing these procedures. Studies have shown that factors such as staff shortage, non-standard environmental conditions, lack of organizational support, and dissatisfaction of nurses negatively affect nursing care and leads to low-quality care (Soltani, 2008).

Baljani and colleagues (2012) found that nurses pay more attention to routine technical aspects of care rather than its psychosocial aspects. Such an approach cannot meet all the patients’ needs. Therefore, such issues should be considered in nursing education and planning programs (Baljani, Azimi, & Hosseinloo, 2012). We found that most nurses were dissatisfied with the fact that they had to spend more time for doing paper work at nursing stations than patients’ bedside.

Meltzer (2004) found that futile care affects professional caregivers. Many studies have addressed problems related to excess treatments for dying patients and their negative effects on the staff. In one study on 759 nurses and 687 physicians, 50% of the nurses, 30% of the physicians, and 70% of home caregivers reported care provision lower than standards (Meltzer, 2004).

## 5. Conclusion

Considering that nurses play a key role in managing futile care, being aware of their experiences in this regard could be the initial operational step to compile useful care giving and educational programs in ICUs. Moreover, the results of this study may help nursing managers adopt supportive approaches to reduce the amount of futile care which could, in turn, resolve some of the complications that nurses face at these wards such as burnout, ethical conflicts, and leave.

**Limitations**

Although the participants shared their experiences with researchers, but this study was conducted in highly specialized units and findings cannot be generalized beyond the population. We suggest that such qualitative research be done in other units and with other health care professionals in Iran.
